# Investigating late‐onset ADHD: a population cohort investigation

**DOI:** 10.1111/jcpp.12911

**Published:** 2018-04-23

**Authors:** Miriam Cooper, Gemma Hammerton, Stephan Collishaw, Kate Langley, Ajay Thapar, Søren Dalsgaard, Evie Stergiakouli, Kate Tilling, George Davey Smith, Barbara Maughan, Michael O'Donovan, Anita Thapar, Lucy Riglin

**Affiliations:** ^1^ MRC Centre for Neuropsychiatric Genetics and Genomics Division of Psychological Medicine & Clinical Neurosciences Cardiff University School of Medicine Cardiff UK; ^2^ Population Health Sciences University of Bristol Bristol UK; ^3^ Cardiff University School of Psychology Cardiff UK; ^4^ National Centre for Register‐based Research School of Business and Social Sciences Aarhus University Aarhus Denmark; ^5^ The Lundbeck Foundation Initiative for Integrative Psychiatric Research, iPSYCH Aarhus Denmark; ^6^ Department for Child and Adolescent Psychiatry Hospital of Telemark Kragerø Norway; ^7^ School of Social and Community Medicine MRC Integrative Epidemiology Unit (IEU) University of Bristol Bristol UK; ^8^ Social, Genetic and Developmental Psychiatry Centre Institute of Psychiatry Kings College London London UK

**Keywords:** Late‐onset ADHD, neurodevelopment, Avon Longitudinal Study of Parents and Children, Strengths and Difficulties Questionnaire

## Abstract

**Background:**

Adult ADHD has been assumed to be a continuation of childhood‐onset ADHD. However, recent studies have identified individuals with ADHD in adulthood who have not had ADHD in childhood. Whether or not these individuals have a ‘typical’ neurodevelopmental profile is not clear.

**Methods:**

We tested two explanations for the emergence of apparent late‐onset ADHD symptomatology using the ALSPAC epidemiological cohort, by grouping individuals according to their scores on the Strengths and Difficulties Questionnaire (SDQ) hyperactivity subscale at ages 12 and 17 years. First, we tested whether some of those with apparent late‐onset ADHD symptoms had been potentially misclassified on the basis of earlier SDQ hyperactivity scores (ages 7, 8 and 9 years) or of subthreshold symptoms at age 12 years. Second, we investigated the possibility that those with ‘genuine’ late‐onset ADHD symptoms had a delayed manifestation of the same liability that underlies childhood‐onset symptoms, by investigating whether they had a similar profile of neurodevelopmental impairments (in the domains of autistic symptomatology, language, reading, spelling, executive functioning and IQ) as those with typical childhood‐onset ADHD.

**Results:**

*N* = 56/75 (75%) of those with apparent late‐onset ADHD had had high ADHD scores at least one point in childhood, suggesting that they may have been misclassified on the basis of their score at age 12 years. The remaining 19 individuals (25%) with genuine late‐onset ADHD symptoms did not show a profile of neurodevelopmental impairment typically seen in ADHD, instead showing similar levels of autistic symptoms, language skills, executive functioning ability and IQ to those without ADHD symptoms. The only exceptions were that this group showed reading and spelling problems at age 9 years.

**Conclusions:**

Our work suggests that this small number of individuals with genuine late‐onset symptoms may not be most appropriately considered as having a typical neurodevelopmental disorder.

## Introduction

Attention deficit hyperactivity disorder (ADHD) is conceptualised by the DSM‐5 (American Psychiatric Association, 2013) as a childhood‐onset neurodevelopmental disorder. Symptoms tend to attenuate with age, although around 60% of individuals have symptom persistence into adulthood and 40% show both symptom persistence and impairment (Sibley, Swanson et al., 2017). Adult ADHD has been assumed to be a continuation of childhood ADHD. Its diagnostic criteria require childhood onset and allow for the developmental change in symptoms over time, with fewer symptoms in each category needing to be endorsed for diagnostic criteria to be met (American Psychiatric Association, 2013).

However, recent findings have challenged the assumption of this trajectory. First, a prospective population study from New Zealand (Moffitt et al., 2015) found that 87% of those with adult ADHD, ascertained by structured interview at age 38 years, had no history of childhood ADHD on the basis of assessments made during childhood. In this study, adult ADHD status was not associated with the male preponderance, neuropsychological deficits or child ADHD genetic liability (as indexed by ADHD polygenic risk scores) typical of childhood onset ADHD, leading the authors to conclude that adult ADHD may not be a continuation of the childhood neurodevelopmental disorder and may have a different aetiology. Second, a Brazilian birth cohort study (Caye et al., 2016) found 85% of their young adult ADHD group, as ascertained at age 18–19 years without requiring the ‘age at onset’ criterion, had not met criteria in childhood (age 11 years). This young adult group had a female preponderance and a range of adverse outcomes including criminality, suicide attempts and comorbidities. Third, a UK twin study (Agnew‐Blais et al., 2016) found that 67% of their young adult ADHD group, as defined at age 18 years again without requiring the ‘age of onset’ criterion, had not met criteria when assessed for ADHD in childhood (at ages 5, 7, 10 and 12 years). Those with late‐onset disorder were more likely to be female, had fewer behaviour problems and higher IQ in childhood than those with persistent ADHD, though were similar in ADHD symptom levels, impairment and mental health comorbidities at age 18 years. However, the findings of these studies have been viewed with caution by some, especially because adult diagnoses, unlike child ADHD, were based primarily on self‐report (Faraone & Biederman, 2016). Thus, it is likely too early to conclude that the adult‐onset ADHD groups are truly distinct from the childhood disorder (Faraone & Biederman, 2016).

Indeed, a very recent analysis seeking to address some of the methodological difficulties associated with the population studies investigating late‐onset ADHD found that after careful scrutiny, 95% of the control group in the Multimodal Treatment of ADHD (MTA) study who initially screened positive on checklists for late‐onset ADHD did not actually meet criteria for a diagnosis, with the occurrence of ADHD symptoms only in the context of heavy substance misuse being the most common reason for this (Sibley, Rohde et al., 2017) and only 2% of the group met criteria for a diagnosis of late‐onset ADHD after excluding cases with subthreshold ADHD symptoms in childhood. Nevertheless, the findings from the three population studies showing apparent ADHD in adulthood without a preceding childhood history cannot be dismissed – the correlates of this presentation need further exploration.

As part of a recent genetic study from our group (Riglin et al., 2016) using a UK birth cohort (Avon Longitudinal Study of Parents and Children, ALSPAC), we identified a group of 122 children (2.5% of the sample) who scored in the abnormal range on the Strengths and Difficulties Questionnaire (SDQ, Goodman, 1997) hyperactivity subscale at age 17 years without having had borderline or abnormal symptom scores at age 7 years [the age of onset cut‐point for ICD‐10 (World Health Organisation, 1992)]. The aim of the current paper was to investigate the characteristics of those with apparent late‐onset ADHD as defined by having high or very high scores on the SDQ hyperactivity subscale at age 17 years, but not prior to or at age 12 years (i.e. the age‐of‐onset criterion for DSM‐5 diagnosis). The data collected at multiple time points in ALSPAC mitigate some, but not all of the methodological difficulties surrounding the cohorts used in the previous analyses. First, the same measures were used throughout childhood and late adolescence, meaning that the emergence of later symptomatology is not purely ascribable to the use of different measures. Second, parent report was available for all time points, as self‐reported ADHD symptoms can be unreliable (Danckaerts, Heptinstall, Chadwick, & Taylor, 1999) and different informants are known to show at best only modest agreement. Also, as was the case in the other cohorts, all measures were obtained prospectively, meaning that recall bias, which can be associated with longitudinal studies of ADHD (Sibley et al., 2012), is not an issue.

We tested two explanations for the emergence of late‐onset ADHD symptomatology:

1. Those with apparent late‐onset ADHD symptoms include individuals who had shown slightly raised or high ADHD symptomatology at least one point in childhood, that is, they have been potentially misclassified on the basis of their score at age 12 years.
2. Late‐onset ADHD symptoms are a delayed manifestation of the same liability that underlies childhood‐onset symptoms – such that those with late‐onset symptoms have a similar profile of neurodevelopmental impairments as those with typical childhood‐onset ADHD (Thapar, Cooper, & Rutter, 2016).

## Methods

### Sample

ALSPAC is a well‐established ongoing prospective longitudinal birth cohort study which started in September 1990. The enrolled core sample consisted of 14,541 mothers living in Avon, England, who had expected delivery dates between April 1, 1991, and December 31, 1992. Of these pregnancies, 13,988 children were alive at 1 year. When the oldest children were aged around 7 years, the sample was increased by *n* = 713 by recruiting eligible families who had not originally joined the study. The resulting total sample size of children who were alive at 1 year was 14,701. Ethical approval for the study was obtained from the ALSPAC Ethics and Law Committee and the Local Research Ethics Committees. All participants gave written informed consent. Full details of the study, measures and sample can be accessed elsewhere (Boyd et al., 2013; Fraser et al., 2013). Please note that the study website contains details of all the data that is available through a fully searchable data dictionary (http://www.bris.ac.uk/alspac/researchers/data-access/data-dictionary). For families with multiple births, we included the oldest sibling. We included individuals in our analyses when primary data on ADHD symptoms were available for at ages 12 and 17 years, (*n* = 4,953).

### ADHD symptom groups

ADHD symptoms were assessed at five time points during childhood and adolescence (roughly ages 7, 8, 9, 12 and 17 years) using the parent‐rated 5‐item SDQ subscale which includes hyperactive and inattentive symptoms (score range 0–10) (Goodman, 1997). In our previous work (Riglin et al., 2016), SDQ hyperactivity subscale scores were classified as normal (scores of 0–5), borderline (6) or abnormal (7–10) as per the SDQ's original 3‐band categorisation. For the current analyses, SDQ hyperactivity scores were classified as close to average (0–5), slightly raised (6–7), high (8) and very high (9–10) using the latest 4‐band classification validated in a large UK community sample (Green, McGinnity, Meltzer, Ford, & Goodman, 2005). We defined ADHD symptom groups based on parent‐rated SDQ hyperactivity subscale scores during childhood (up to and including age 12 years, as per DSM‐5 age‐of‐onset criterion for ADHD diagnosis) and adolescence (age 17 years) as follows. Individuals were initially categorised based on whether they scored low (close to average or slightly raised score, i.e. 0–7) or high (i.e. 8–10) at ages 12 and 17 years into one of four ADHD groups: low symptoms, childhood‐limited, childhood‐onset persistent and apparent late‐onset. The apparent late‐onset group was then divided into: (a) potential misclassification – individuals with elevated (slightly raised, high or very high, i.e. 6–10) scores at ages 7, 8 or 9 years, or with a slightly raised score (i.e. 6–7) at age 12 years i.e. individuals with subthreshold/high symptoms at an earlier point in childhood and/or subthreshold symptoms at age 12 years, and (b) genuine late‐onset – those with close to average scores at every time point in childhood (ages 7–12 years). The categorisation of ADHD symptom groups is shown in Table 1.

**Table 1:** Categorisation of ADHD groups according to SDQ hyperactivity subscale scores at different time points

	Low symptoms	Childhood‐limited	Childhood‐onset persistent	Apparent late‐onset	
SDQ score age 12 years	Close to average or slightly raised (0–7)	High or very high (8–10)	High or very high (8–10)	Close to average or slightly raised (0–7)	*Misclassification* Slightly raised (6–7), high (8) or very high score (9–10) at ages 7, 8, or 9 years, or slightly raised score (6–7) at age 12 years *Genuine late‐onset* Close to average score (0–5) at ages 7, 8, 9 and 12 years
SDQ score age 17 years	Close to average or slightly raised (0–7)	Close to average or slightly raised (0–7)	High or very high (8–10)	High or very high (8–10)	

### Family income, emotional, behavioural and social problems, and substance misuse

The following variables were examined for descriptive purposes. Family income was classified as below average or above average using a (within‐sample) median split. Scores on the SDQ emotional problems, conduct problems, prosocial behaviour and peer problems subscales were used to describe difficulties in these domains at ages 12 and 17 years. All SDQ subscales are scored out of 10; higher scores represent greater impairment except for prosocial behaviour, where lower scores represent lower levels of prosocial traits which reflects more impairment (Goodman, 1997). The self‐report 10‐item Alcohol Use Disorders Identification Test (Babor, Higgins‐Biddle, & Saunders, 2001), which is a brief screening tool to identify individuals with alcohol‐related problems, was completed by the young person at approximately age 17 years via postal questionnaire. The AUDIT scale has been studied extensively and has high validity and reliability in the detection of risky drinking, alcohol misuse and alcohol dependence (Allen, Litten, Fertig, & Babor, 1997). Cannabis use was assessed in the same postal questionnaire with the question ‘Have you ever tried cannabis (also called marijuana, hash, dope, pot, skunk, puff, grass, draw, ganja, spliff, joints, smoke, weed)’.

### Neurodevelopmental characteristics

Autistic traits were measured at age 8 years using the Social Communication Disorders Checklist (Skuse, Mandy, & Scourfield, 2005), a parent‐rated 12‐item scale assessing abnormalities in the domains of reciprocal social interaction and communication, with each item being scored from 0–2 and higher scores representing higher levels of autistic traits. Pragmatic language difficulties, intelligibility/fluency and syntax were assessed at age 9 years using the Children's Communication Checklist 2 (Norbury, Nash, Baird, & Bishop, 2004), a parent‐rated questionnaire assessing various aspects of communication, with lower scores representing greater impairment. Reading and spelling were assessed at age 9 years by asking the child to read 10 (real) words selected from previous research (Nunes, Bryant, & Olsson, 2003) and to spell 15 words. IQ was assessed at age 8 years using the Wechsler Intelligence Scale for Children [WISC‐IV, (Wechsler, 2003)], which assesses an age‐scaled full‐scale IQ comprising 10 subtests contributing to four cognitive domains: verbal comprehension index (VCI), perceptual reasoning index (PRI), working memory index (WMI), and processing speed index (PSI). Executive functioning was measured using the digit span score from the WISC‐IV, in which lower scores reflect poorer executive functioning. IQ was measured at age 16 years using the two‐subtest form of the Wechsler Abbreviated Scale of Intelligence (WASI, Wechsler, 1999).

### Analysis strategy

First, we identified the number of individuals with apparent late‐onset symptoms. To test hypothesis 1, we examined how many of these were subsequently categorised as cases of potential misclassification.

For descriptive purposes, groups were compared on family income, emotional, behavioural and social problems, and substance misuse problems.

To test our second hypothesis, we compared the genuine late‐onset group to (a) the low symptoms group (i.e. for those without childhood symptoms, comparing those with to those without adolescent symptoms) and (b) the childhood‐onset persistent group (i.e. for those with adolescent symptoms, comparing those without to those with childhood symptoms) on a range of neurodevelopmental characteristics, using logistic/linear regression as appropriate. The groups were compared on established indicators of neurodevelopmental risk and symptomatology known to be associated with ADHD (Thapar, Cooper, Eyre, & Langley, 2013; Thapar & Rutter, 2015; Thapar et al., 2016) – namely autistic traits, pragmatic language, intelligibility/fluency and syntax, reading and spelling difficulties, executive functioning and IQ. These analyses were to illustrate differences in the level of neurodevelopmental symptomatology between the groups, not to examine a direction of effect in relation to ADHD symptom group membership. Scores on the SDQ, SCDC, CCC‐2 were prorated if <30% of data were missing.

Our primary analyses were conducted on an imputed dataset based on cases with complete data on ADHD symptoms at ages 12 and 17 years, (*n* = 4,953). Of these individuals, the number of respondents with complete data on other variables in the analysis ranged from 3,383 (for adolescent IQ) to 4,953 (for child sex). Missing data were imputed using multivariate imputation by chained equations (Van Buuren & Oudshoorn, 2000), which assumes that data are missing‐at‐random (MAR; i.e. given the observed data included in the imputation model, the missingness mechanism does not depend on the unobserved data (White, Royston, & Wood, 2011). Missing data were found to be dependent on several observed variables available for the full cohort at birth or the early postnatal period (such as family income, maternal education, financial hardship, and parental mental health and substance use); therefore, these were included in the imputation model to make the assumption of MAR as plausible as possible. In addition, the imputation model included all variables included in the analysis models, and a number of auxiliary variables that were strongly associated with the measures to be imputed (such as multiple measures of child substance use, cognition, communication skills and mental health at other ages). Using binary and multinomial logistic and linear regression models as appropriate, 100 imputed datasets were derived, each with 10 cycles of regression switching. Predictive mean matching was used when continuous variables were not normally distributed. All analyses were then run on imputed datasets by combining estimates using Rubin's rules (White et al., 2011) using STATA version 15. Sensitivity analyses conducted on the complete data (without imputation) are presented in the supporting information in the online version of this article.

## Results

Of 4,953 individuals with SDQ hyperactivity data at ages 12 and 17 years, *n* = 4,692 (94.7%) were categorised in the low symptoms group, *n* = 119 (2.4%) were in the childhood‐limited group, *n* = 55 (1.1%) were in the childhood‐onset persistent group and *n* = 87 (1.7%) were in the apparent late‐onset group.

### Hypothesis 1

**Figure 1:**
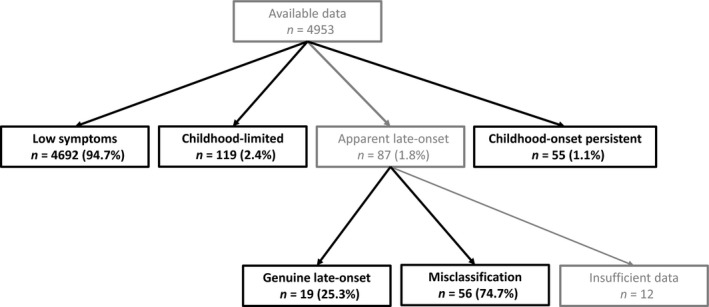
Categorising individuals based on ADHD symptoms

Of the 87 individuals with apparent late‐onset symptoms, *n* = 12 (13.8%) had insufficient data prior to age 11/12 years to establish whether they belonged to the misclassification or genuine late‐onset group (*n* = 12). Of the *n* = 75 with sufficient data, *n* = 56 (74.7%) were categorised as potential misclassification (based on the presence of slightly raised/high symptoms on at least one childhood time point), with *n* = 19 (25.3%) categorised as genuine late‐onset. A flowchart demonstrating the numbers in the groups is shown in Figure 1.

A total of 44.1% of the low symptoms group, 63.3% of the persistent group and 51.1% of the genuine late‐onset group were classified as below the median family income [genuine late‐onset vs. low symptoms OR = 1.32 (95% CI: 0.44, 3.92), *p* = .620; genuine late‐onset vs. childhood‐onset persistent OR = 0.60 (95% CI: 0.18, 2.09), *p* = .425].

**Figure 2:**
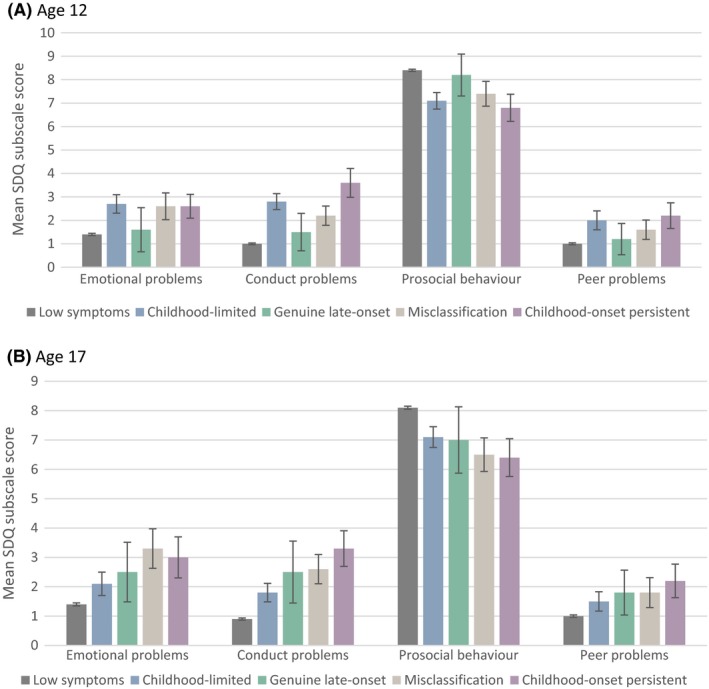
Emotional, behavioural and social problems as assessed by mean SDQ subscale scores, by ADHD symptom group. A) at age 12 years, B) at age 17 years. SDQ, Strengths and Difficulties Questionnaire. Error bars = 95% confidence intervals

Figure 2 shows comparisons of each of the ADHD symptom groups on the SDQ subscales. As shown in Table 2, the genuine late‐onset group had lower comorbid symptom levels at age 12 years than the childhood‐onset persistent group. However, at age 17 they had more comorbid symptoms than the low symptoms group but did not differ from the childhood‐onset persistent group for emotional problems, peer problems or prosocial behaviour.

**Table 2:** Comparisons of the ADHD symptom groups on emotional, behavioural and social problems as assessed by SDQ subscale scores

	Genuine late‐onset vs. low symptoms		Genuine late‐onset vs. childhood‐onset persistent	
	Mean difference (95% CI)	*p*	Mean difference (95% CI)	*p*
Childhood: age 12				
Emotional problems	0.21 (−0.54, 0.96)	.585	−1.00 (−1.87, −0.13)	.024
Conduct problems	0.50 (−0.08, 1.08)	.091	−2.06 (−2.73, −1.39)	<.001
Prosocial behaviour	−0.22 (−0.95, 0.51)	.559	1.37 (0.53, 2.22)	.001
Peer problems	0.14 (−0.54, 0.82)	.691	−1.08 (−1.86, −0.29)	.008
Adolescence: age 17				
Emotional problems	1.05 (0.20, 1.91)	.016	−0.60 (−1.59, 0.38)	.227
Conduct problems	1.52 (0.92, 2.11)	<.001	−0.92 (−1.61, −0.23)	.009
Prosocial behaviour	−1.01 (−1.85, −0.17)	.019	0.74 (−0.24, 1.72)	.141
Peer problems	0.68 (0.02, 1.35)	.044	−0.56 (−1.34, 0.22)	.160

NB. Mean scores for the childhood‐onset persistent group were higher than for the low symptoms group on all variables at *p* < .001.

There was weak evidence that the mean score for alcohol use was higher for the genuine late‐onset compared to the low symptoms group [mean = 10.04 vs. 6.27: mean difference = 3.76 (95% CI: −0.07, 7.60) *p* = .054]; however, there was little evidence that it differed from the childhood‐onset persistent group [mean = 7.23: mean difference = 2.81 (95% CI: −1.43, 7.05) *p* = .193].

Finally, 28.1% of the low symptoms group, 32.9% of the persistent group and 52.8% of the genuine late‐onset group reported cannabis use by age 17 years [genuine late‐onset vs. low symptoms OR = 2.87 (95% CI: 0.93, 8.91), *p* = .067; genuine late‐onset vs. childhood‐onset persistent OR = 2.29 (95% CI: 0.60, 8.83), *p* = .225].

### Hypothesis 2

As shown in Table 3, the genuine late‐onset group showed similar scores to the low symptoms group on neurodevelopmental features which are associated with ADHD. The exception was that the genuine late‐onset group showed poorer reading and spelling at age 9 years compared to the low symptoms group, with scores that were comparable to those of the childhood‐onset persistent group.

**Table 3:** Comparisons of the ADHD symptom groups on neurodevelopmental characteristics and educational attainment

	Low symptoms (*N* = 4,692)	Childhood‐limited (*N* = 119)	Genuine late‐onset (*N* = 19)	Misclassification (*N* = 56)	Childhood‐onset persistent (*N* = 55)	Genuine late‐onset vs. low symptoms		Genuine late‐onset vs. childhood‐onset persistent	
Male gender (%)	47.7%	63.9%	63.2%	60.7%	80.0%	χ^2^(1) = 1.81, *p* = .179		χ^2^(1) = 2.18, *p* = .140	
	Mean (*SE*)					Mean difference (95% CI)	*p*	Mean difference (95% CI)	*p*
Autistic symptoms	2.4 (0.5)	7.2 (0.5)	2.7 (0.7)	6.4 (0.7)	9.7 (0.8)	0.28 (−1.24, 1.79)	.719	−6.98 (−8.75, −5.21)	<.001
Pragmatic language	151.9 (0.1)	142.6 (1.0)	152.2 (1.0)	144.9 (1.3)	137.5 (1.5)	0.39 (−2.80, 3.58)	.809	14.71 (11.02, 18.41)	<.001
Intelligibility/fluency	35.4 (0.03)	34.8 (0.2)	35.8 (0.2)	35.1 (0.3)	34.0 (0.5)	0.39 (−0.41, 1.19)	.336	1.79 (0.86, 2.73)	<.001
Syntax	31.9 (0.01)	31.5 (0.1)	32.0 (0.0)	31.7 (0.1)	31.4 (0.2)	0.12 (−0.10, 0.35)	.268	0.55 (0.29, 0.81)	<.001
Reading	7.8 (0.3)	6.3 (0.3)	6.5 (0.6)	7.5 (0.3)	5.5 (0.5)	−1.31 (−2.38, −0.24)	.017	0.98 (−0.25, 2.22)	.119
Spelling	10.6 (0.5)	8.3 (0.4)	8.8 (0.8)	10.5 (0.4)	7.3 (0.6)	−1.80 (−3.34, −0.25)	.023	1.50 (−0.28, 3.29)	.099
Executive functioning	10.6 (0.5)	9.0 (0.3)	10.1 (0.7)	9.9 (0.5)	9.6 (0.5)	−0.48 (−1.95, 1.00)	.527	0.49 (−1.22, 2.19)	.576
Childhood IQ	107.0 (0.3)	98.4 (1.7)	102.4 (3.6)	102.7 (2.1)	95.0 (2.4)	−4.55 (−12.02, 2.92)	.232	7.45 (−1.23, 16.13)	.092
Adolescent IQ^a^	93.2 (0.2)	87.2 (1.4)	90.3 (3.6)	90.0 (2.0)	87.1 (1.9)	−2.85 (−9.49, 3.79)	.401	3.21 (−4.57, 10.98)	.419

Mean scores differed between the low symptoms and childhood‐onset persistent groups on all variables at *p* < .05.

a Note different mean scores between childhood and adolescence due to the use of different measures.

## Discussion

In this UK longitudinal population‐based cohort, using updated SDQ cut‐points and excluding those with high scores at one time point during childhood, we find lower rates of genuine late‐onset ADHD (0.4%) than other population‐based studies (Moffitt et al., 2015, 2.7%; Agnew‐Blais et al., 2016, 5%; Caye et al., 2016, 7.9%). Indeed, in our sample, 75% (56 of 75 with full data) of those with apparent late‐onset ADHD symptoms were due to potential misclassification once we had taken into account subthreshold or elevated SDQ ADHD scores at least one point in childhood. This is in keeping with research suggesting that reported ADHD symptom levels and ADHD subgroup membership (i.e. inattentive, hyperactive‐impulsive) can fluctuate over time (Willcutt et al., 2012). Moffitt et al. (2015) found that their 3% prevalence of adult ADHD at age 38 years was 90% comprised of those without a history of childhood ADHD; Caye et al. (2016) found that 84.6% of their 18/19 year olds with ADHD did not have a history of childhood ADHD, and Agnew‐Blais et al. (2016) found that 67.5% of their 18 year olds with ADHD had not met criteria for ADHD at any assessment in childhood. Our higher rates of individuals with apparent late‐onset symptoms who we then considered to actually have a childhood history of significant ADHD symptoms (i.e. those who we are considering as misclassified) may be explained by the availability of multiple assessment points in childhood. Nevertheless, in keeping with other cohort studies, we do identify a remaining group of what may be genuinely late‐onset ADHD.

Thus, our first hypothesis is only partially supported – a majority of late‐onset ADHD could be explained by misclassification, but this does not account for the entire group. We cannot infer anything clinically relevant about the nature of the misclassification group as it is a heterogeneous group which includes, for example, those who had ‘episodically’ raised ADHD symptoms at disparate points in childhood and those who had subthreshold symptoms at age 12 years. However, we believe that the larger number of individuals in the misclassification group, compared to the much smaller stringently defined genuine late‐onset group, is a notable finding.

The richness of the ALSPAC dataset allows the first detailed investigation of neurodevelopmental and neurocognitive factors in relation to late‐onset ADHD. Our second hypothesis was that late‐onset ADHD symptoms are a delayed manifestation of the same liability that underlies childhood‐onset ADHD symptoms. We hypothesised that if this was the case, the genuine late‐onset group would share similarities with typical childhood‐onset ADHD on measures that characterise the childhood neurodevelopmental disorders (that include autism spectrum disorder, communication disorders, intellectual disability, motor disorders and specific learning disorders (American Psychiatric Association, 2013, Thapar et al., 2016). ADHD and other neurodevelopmental disorders typically show strong overlap with each other and are characterised by neurocognitive deficits such as impaired executive function (Thapar & Rutter, 2015; Thapar et al., 2016). Our results showed that the remaining genuine late‐onset group did not show a neurodevelopmental profile characterised by features or impairment that are typical of childhood‐onset ADHD, and their childhood neurodevelopmental profile was more similar to the low symptoms group than to the childhood‐onset persistent group in this regard. Intriguingly, the one exception was that the genuine late‐onset group showed lower scores on reading and spelling measures at 9 years. This was not accompanied by language problems. We speculate that children with reading/spelling difficulties at age 9 years do not show mental health problems at this age but as they move through the educational system, they encounter higher academic demands and start to develop multiple mental health problems (including ADHD‐like symptoms), substance use and peer difficulties through adolescence.

In line with our results, the adult ADHD group of Moffitt et al. (2015) did not show a deficit in IQ (with a mean IQ of 96.94) and the young adult group of Agnew‐Blais et al. (2016) had a mean IQ of 96.12 which was significantly higher than that of their persistent group (89.78). Interestingly, the percentage of our genuine late‐onset group with a household income below the (within‐group) median was 51%, suggesting that this group does not show socioeconomic disadvantage, a factor which is associated with childhood ADHD (Russell, Ford, & Russell, 2015). However, this group did still show a male excess, with 63% of the group being male, though this excess was not as pronounced as that in the ‘childhood‐onset’ group (80%).

Some have questioned whether adolescent‐onset ADHD symptomatology at age 17 years represents the emergence of a mood disorder or conduct problems, or a substance misuse disorder (Caye, Sibley, Swanson, & Rohde, 2017; Sibley, Rohde et al., 2017). The difficulty with investigating these factors is that concurrent and sequential comorbidity including substance misuse typifies childhood‐onset ADHD (Barkley, Fischer, Smallish, & Fletcher, 2004; Chang, Lichtenstein, & Larsson, 2012) so in our view cannot be used to distinguish late‐onset ADHD from childhood‐onset ADHD. However, in the interests of characterising the group we described symptoms of comorbid mental health problems. Intriguingly, within the genuine late‐onset group, difficulties on each of the SDQ subscales increased between the age of 12 years (when they did not appear to have emotional, behavioural or social problems) and 17 years, when they showed scores more comparable with the childhood‐onset persistent group. This is in contrast to the relatively stable SDQ scores over time in both the low symptoms and childhood‐onset persistent groups. Levels of alcohol use and rates of cannabis use were higher in the genuine late‐onset group than in both the low symptoms and childhood‐onset persistent groups, but the observed associations do not appear strong enough to explain the presence of late‐onset ADHD (e.g. nearly half the genuine late‐onset group did not report having ever used cannabis). We cannot determine the direction of the association between late‐onset symptoms of ADHD and symptoms of other mental health problems, or what might explain this change, beyond a possible influence of substance use. Having said this, we found that the levels of difficulties in the SDQ domains were not as high as in the childhood‐onset persistent group. Thus, in this population‐based cohort, we conclude that comorbid mental health and substance misuse problems are unlikely to fully account for the emergence of late‐onset ADHD symptomatology.

Previous studies have found conflicting results in this regard, with Moffitt et al. (2015) not finding a link between childhood or adult ADHD status and mood or anxiety problems, Agnew‐Blais et al. (2016) not finding elevated rates of depression and anxiety in their late‐onset ADHD group compared to the persistent group, but Caye et al. (2016) finding elevated rates of mood disorders and anxiety in their young adult ADHD group. Moffitt et al. (2015) found that their adult ADHD group had persistently elevated rates of substance dependence, as did the late‐onset and persistent ADHD groups of Agnew‐Blais et al. (2016). On the contrary, elevated rates of substance dependence were not found in the young adult ADHD group of Caye et al. (2016). Our investigation was conducted in a younger age group than some of the previous studies and before the peak age of onset for mood disorders (Kessler et al., 2007) (but not conduct disorder) so the age difference might be one contributory factor.

### Strengths and limitations

As highlighted at the outset of this paper, notable strengths are the use of the same ADHD assessment measures throughout childhood and adolescence, the availability of parent report at all time points, and the prospective nature of all measures, as well as the detailed neurodevelopmental and cognitive phenotype data available in ALSPAC. We have also used multiple imputation to avoid the biases associated with missing data. It is also worth mentioning that of the three previous epidemiological studies investigating whether adult ADHD is preceded by childhood ADHD, the adult ADHD groups of Caye et al. (2016) and Moffitt et al. (2015) do not specifically distinguish those without a childhood history when examining phenotypic associations with group membership, with only Agnew‐Blais et al. (2016) identifying a pure late‐onset group.

Limitations of our study include the small size of the genuine late‐onset group, which limits statistical power to detect associations with the variables under study. Second, as is the case for any longitudinal study, there was selective attrition over time; however, potential bias arising from missing data was dealt with using multiple imputation, utilising a large amount of additional information to make the assumption of missing‐at‐random as plausible as possible. Findings from analyses using imputed data and from complete data were very similar. Nevertheless, selective attrition of those likely to have the highest ADHD symptomatology is likely (Hay, McStephen, Levy, & Pearsall‐Jones, 2002) and would have affected our analytic sample (i.e. those with ADHD data). Indeed, the childhood neurodevelopmental characteristics that we assessed were associated with (not) being in our analytical sample (see Table S3), which may have affected our rates of late‐onset symptoms and led to underestimated rates of accompanying problems. Third, our use of a screening instrument for defining the groups will have resulted in different exclusions to those using diagnostic instruments, which has been raised as a limitation of this type of research (Caye et al., 2017). However, given that ADHD symptoms act as a continuously distributed dimension in terms of associations with risk factors and outcomes (Thapar & Cooper, 2016) and our ADHD measure at age 17 years remained based on the same informant at all time points (parent report) as opposed to switching from parent to self‐report our investigation has many strengths as well as limitations. Nevertheless, assessment issues aside, we do still identify a group of those with late‐onset ADHD in keeping with a previous report using a less stringent cut‐point in this same data set (Riglin et al., 2016) as well as other studies (Agnew‐Blais et al., 2016; Caye et al., 2016; Moffitt et al., 2015; Sibley, Rohde et al., 2017).

### Summary and clinical relevance

In summary, 75% of those with apparent late‐onset symptoms were due to potential misclassification in childhood. The remaining genuine late‐onset group did not show a characteristic neurodevelopmental profile with the only exception being lower scores on spelling and reading measures in middle childhood. However, they showed elevated scores on measures of behavioural, emotional, social and peer problems by late adolescence, and a trend towards higher levels of alcohol and cannabis use.

The existence of this group is of clinical significance as it implies that there may be a small number of individuals who will present with ADHD symptoms in late adolescence without either a childhood history of inattention or hyperactivity (currently required for diagnosis) or the profile of neurodevelopmental and social adversity often associated with ADHD. These individuals need further characterisation in the context of both epidemiological and possibly clinic‐based and treatment studies. Our work suggests that they may not be most appropriately considered as having a neurodevelopmental disorder and a crucial issue is whether treatments currently recommended for ADHD are effective in this group. Due to the transitional age of the individuals involved, this has implications for practice in both child and adolescent mental health services and adult psychiatric services.

**Key points**

- ADHD is currently conceptualised as a childhood‐onset neurodevelopmental disorder.
- Recent research has challenged this assumption, with several longitudinal studies finding evidence for a group of individuals with ADHD symptoms arising for the first time after childhood.
- Using the ALSPAC dataset, we have identified a small group of individuals with ADHD symptoms arising in late adolescence who do not have a profile of neurodevelopmental impairments typical of ADHD.
- Our work suggests that this group may not be most appropriately considered as having a neurodevelopmental disorder, and a crucial issue for further exploration is whether treatments currently recommended for ADHD are effective in this group.

## Supporting information


**Table S1.** Comparisons of the ADHD symptom groups on emotional, behavioural and social problems using complete cases.
**Table S2.** Comparisons of the ADHD symptom groups on neurodevelopmental characteristics and educational attainment using complete cases.
**Table S3.** Association with sample retention.
