# Supplementary material for: Investigating late‐onset ADHD: a population cohort investigation
Source: J Child Psychol Psychiatry. 2018 Apr 23;59(10):1105–13. doi: 10.1111/jcpp.12911 (PMC6175329; doi:10.1111/jcpp.12911)
Supplement: Supplementary file 1 — Table S1. Comparisons of the ADHD symptom groups on emotional, behavioural and social problems using complete cases. Table S2. Comparisons of the ADHD symptom groups on neurodevelopmental characteristics and educational attainment using complete cases. Table S3. Association with sample retention. [file JCPP-59-1105-s001.docx]

**Additional Supporting Information for: Investigating late-onset ADHD: A population cohort investigation – by Miriam Cooper et al.**

**Supplementary Table S1.** Comparisons of the ADHD symptom groups on emotional, behavioural and social problems using complete cases.

|  | Genuine late-onset  vs. low symptoms | | | Genuine late-onset  vs. childhood-onset persistent | | |
| --- | --- | --- | --- | --- | --- | --- |
|  | Mean difference (95% CI) | | p | Mean difference (95% CI) | | p |
| Childhood: age 12 |  |  |  |  |  |  |
| Emotional problems | 0.21 | (-0.54, 0.96) | 0.582 | -1.00 | (-1.87, -0.13) | 0.024 |
| Conduct problems | 0.50 | (-0.07, 1.08) | 0.088 | -2.06 | (-2.72, -1.39) | <0.001 |
| Prosocial behaviour | -0.22 | (-0.96, 0.51) | 0.549 | 1.37 | (0.53, 2.22) | 0.001 |
| Peer problems | 0.17 | (-0.52, 0.85) | 0.636 | -1.02 | (-1.80, -0.23) | 0.011 |
| Adolescence: age 17 |  |  |  |  |  |  |
| Emotional problems | 1.10 | (0.25, 1.96) | 0.011 | -0.57 | (-1.54, 0.41) | 0.255 |
| Conduct problems | 1.59 | (1.00, 2.18) | <0.001 | -0.78 | (-1.46, -0.10) | 0.024 |
| Prosocial behaviour | -1.09 | (-1.93, -0.24) | 0.012 | 0.64 | (-0.34, 1.61) | 0.204 |
| Peer problems | 0.75 | (0.08, 1.41) | 0.028 | -0.42 | (-1.19, 0.35) | 0.287 |

NB. Mean scores for the childhood-onset persistent group were higher than for the low symptoms group on all variables at p<0.001

**Supplementary Table S2.** Comparisons of the ADHD symptom groups on neurodevelopmental characteristics and educational attainment using complete cases.

|  | Low  symptoms (N=4692)^a^ | Childhood-limited  (N=119)^b^ | Genuine  late-onset  (N=19)^c^ | Misclass-ification  (N=56)^d^ | Childhood-onset persistent (N=55)^e^ | Genuine late-onset  vs. low symptoms | | | Genuine late-onset  vs. childhood-onset persistent | | |
| --- | --- | --- | --- | --- | --- | --- | --- | --- | --- | --- | --- |
| Male gender (%) | 47.7% | 63.9% | 63.2% | 60.7% | 80.0% | χ^2^(1)=1.81, p=0.179 | | | χ^2^(1)=2.18, p=0.140 | | |
|  | Mean (SE) | | | | | Mean difference (95% CI) | | p | Mean difference (95% CI) | | p |
| Autistic symptoms | 2.4 (0.5) | 7.2 (0.6) | 2.6 (0.7) | 6.4 (0.8) | 9.6 (0.8) | 0.25 | (-1.28, 1.78) | 0.749 | -6.97 | (-8.73, -5.21) | <0.001 |
| Pragmatic language | 151.9 (0.1) | 143.2 (0.9) | 152.3 (0.9) | 145.0 (1.4) | 137.6 (1.5) | 0.34 | (-2.87, 3.54) | 0.835 | 14.69 | (11.00, 18.37) | <0.001 |
| Intelligibility/fluency | 35.4 (0.0) | 34.9 (0.2) | 35.8 (0.2) | 35.1 (0.3) | 34.0 (0.5) | 0.39 | (-0.41, 1.19) | 0.337 | 1.77 | (0.84, 2.70) | 0.002 |
| Syntax | 31.9 (0.0) | 31.6 (0.1) | 32.0 (0.0) | 31.2 (0.1) | 31.5 (0.2) | 0.12 | (-0.10, 0.34) | 0.269 | 0.55 | (0.29, 0.80) | <0.001 |
| Reading | 7.8 (0.0) | 6.4 (0.3) | 6.6 (0.6) | 7.6 (0.3) | 5.7 (0.5) | -1.29 | (-2.34, -0.24) | 0.016 | 0.85 | (-0.38, 2.07) | 0.176 |
| Spelling | 10.7 (0.1) | 8.4 (0.4) | 8.9 (0.8) | 10.7 (0.4) | 7.7 (0.7) | -1.78 | (-3.29, -0.27) | 0.021 | 1.23 | (-0.55, 3.01) | 0.175 |
| Executive functioning | 10.7 (0.0) | 9.1 (0.3) | 10.2 (0.7) | 10.0 (0.5) | 9.7 (0.5) | -0.46 | (-1.95, 1.03) | 0.546 | 0.45 | (-1.28, 2.18) | 0.611 |
| Childhood IQ | 107.5 (0.3) | 99.7 (1.8) | 102.5 (3.6) | 103.2 (2.1) | 95.0 (2.5) | -4.95 | (-12.39, 2.49) | 0.192 | 7.50 | (-1.20, 16.20) | 0.091 |
| Adolescent IQ | 93.6 (0.2) | 86.4 (1.6) | 93.2 (4.2) | 90.4 (2.3) | 87.8 (1.9) | -0.46 | (-7.71, 6.80) | 0.902 | 5.33 | (-2.86, 13.52) | 0.202 |

Sample size minimum (for adolescent IQ) and maximum (for gender) values: ^a^3204-4692, ^b^78-119, ^c^12-19, ^d^38-56, ^e^43-55. Mean scores differed between the low symptoms and childhood onset persistent groups on all variables at p<0.05.

**Supplementary Table S3.** Association with sample retention.

|  | OR | (95% CI) | p |
| --- | --- | --- | --- |
| Male gender (N=14498) | 1.17 | (1.10-1.26) | <0.001 |
| Income* (N=6471) | 0.86 | (0.85-0.88) | <0.001 |
| Autistic symptoms (N=8015) | 1.03 | (1.02-1.05) | <0.001 |
| Pragmatic language (N=7822) | 0.97 | (0.96-0.97) | <0.001 |
| Intelligibility/fluency (N=8046) | 0.94 | (0.92-0.96) | <0.001 |
| Syntax (N=8018) | 0.77 | (0.71-0.83) | <0.001 |
| Reading (N=7534) | 0.90 | (0.89-0.92) | <0.001 |
| Spelling (N=7528) | 0.93 | (0.92-0.94) | <0.001 |
| Executive functioning (N=7103) | 0.94 | (0.93-0.95) | <0.001 |
| Childhood IQ (N=7243) | 0.97 | (0.97-0.98) | <0.001 |

Total possible N=14498: predicting membership in our analytic sample (N=4953)

* Income assessed as the average household income band, including social benefits, each week when the child was on a ten-point scale when the child was 134 months old.
